# Physical Activity, Blood Glucose and C-Peptide in Healthy School-Children, a Longitudinal Study

**DOI:** 10.1371/journal.pone.0156401

**Published:** 2016-06-07

**Authors:** Karina Huus, Linda Åkerman, Anders Raustorp, Johnny Ludvigsson

**Affiliations:** 1 CHILD research group, Department of Nursing, School of Health and Welfare, SE- 551 11 Jönköping, Sweden; 2 Division of Pediatrics, Department of Clinical and Experimental Medicine, Linköping University, SE- 58185 Linköping, Sweden; 3 Linnaeus University, School of Sport Sciences, SE-39182 Kalmar, Sweden; 4 Department of Nutrition and Sport Sciences, University of Gothenburg, SE-40530 Gothenburg, Sweden; 5 Division of Pediatrics, Department of Clinical and Experimental Medicine, Linköping University, SE- 58185 Linköping, Sweden; 6 Pediatric Clinic, Östergötland, County Council, SE -58185 Linköping, Sweden; Medical University Innsbruck, AUSTRIA

## Abstract

**Aim:**

To further elucidate the relationship between physical activity and several risk factors for development of diabetes (glucose, C-peptide and obesity) over time.

**Methods:**

A prospective longitudinal study where physical activity was measured on 199 children from Kalmar and Linköping at age 8, and the same 107 children from Linköping again at age 12. Anthropometric data was collected and blood was analyzed for C-peptide and f-glucose. The children in the study were representative for the general Swedish child population, and on an average lean.

**Results:**

High physical activity was related to lower C-peptide at age 8 and 12. This correlation was especially pronounced in boys, who also were more physically active than girls at both time points. The association seen at 8 years of age was similar at age 12 in most children. Children with higher BMI Z-Score had a higher fasting C-peptide (age 12) but linear regression showed that children with more steps per day were less likely to have a higher fasting C-peptide irrespective of BMI. Longitudinal follow-up showed that a decrease in physical activity increased insulin resistance and β-cell load.

**Conclusions:**

Already in young children, physical activity improves insulin sensitivity and decreases the need of C-peptide over time. This seems to become even more pronounced with increasing age when children are followed longitudinally. Low physical activity increases the load on insulin producing β-cells, might increase the risk for both type 1- and 2 diabetes.

## Introduction

According to a recent report, the prevalence of overweight/obesity among 7–9 year olds in Sweden is 17% [1]. This is a high figure, although the increase in body weight among children now may have reached a plateau in Sweden and some other countries [2]. Even moderately elevated body mass index (BMI) has a negative impact on metabolic profile and cardiovascular risk factors in children [3], and increased body weight among children has also been suggested as one factor possibly contributing not only to type 2 diabetes but also to the increased incidence of type 1 diabetes (T1D) [4–6]. Low physical activity is associated with obesity and with cardio metabolic risk in children [7–10]. A central element in the *accelerator hypothesis [4]*, the *β-cell stress hypothesis [5]* and the *overload hypothesis* [6] is that excessive activity of pancreatic β-cells (due to for example extra body weight) either causes or accelerates the β-cell destruction that is characteristic of T1D. In support of these theories, it has been shown that children who later develop T1D have a greater weight gain in early childhood compared to children who remain non-diabetic [11–13], and that BMI is inversely correlated to age of disease onset [14,15]. Earlier studies show that the glucose-stimulated insulin secretion normally is increased during puberty, a response that may be a compensation for increased insulin resistance during puberty [16,17]. Insulin resistance and increased insulin secretion has been found early before development of diabetes [18].

The aim of this study was to further elucidate the relationship between physical activity and several risk factors for development of diabetes (glucose, C-peptide and obesity) over time.

## Materials and Methods

### Subjects and study design

This is a longitudinal study, where the same cohort of children was followed prospectively as part of the large ABIS-cohort (All Babies In southeast Sweden)[19]. Children from different school classes were asked to participate in an assessment of physical activity and glucose homeostasis. The school nurse asked the children and both their parents to give written informed consent before participation. All children who were asked wanted to participate. Inclusion criteria were age group 8 and 12 years and going in the selected schools. The only exclusion criterion was severe handicap making it impossible to wear a pedometer. The chosen schools were shown to be representative for ABIS children in the same area, with regard to relevant factors reflecting physical activity (Table 1), and ABIS children are in turn representative for the general population of children in Sweden.

**Table 1:** Parent-reported average time spent in activities relevant for level of physical activity in children participating in the present study and in all ABIS-children in the same area.

	Present study, age 8 (n = 152)	ABIS children from the same area, age 8 (n = 3962)
Girls/ boys	48% / 52%	51% / 49%
Hours spent outdoors on schooldays	4.72	4.54
Hours spent in front of computer	1.53	1.69
Hours spent in physical activity	5.48	5.40
Hours spent reading/doing homework	2.04	2.05

The study size was based on earlier knowledge on number of daily steps in these age groups and the variation in number of steps among children. A standard deviation (SD) of 3000 steps estimated that a number of 84 children should give enough power (0.80), but in the first measurement we doubled the size by studying children from two different parts of the ABIS-region, Linköping and Kalmar. Participants wore pedometers to measure the average number of daily steps, and blood samples were collected. Weight, height and waist circumference were recorded. The children were around 8 years old (mean 7.8 years, range 6.5–8.9) at the first measurement. The procedure was repeated 4 years later when these children were around 12 years old (mean 11.9, range 10.6–12.6), but this follow-up could only be performed in Linköping for practical reasons. Lack of personal resources at the Kalmar site made it impossible to collect pedometer data. Self-reported data on variables connected to physical activity, from parents and from the children themselves, were available from questionnaires at the same time points.

The study has been approved by the Research Ethics Committee at the Faculty of Health Sciences, Linköping University, Sweden (Dnr 36287 and Dnr 03–092).

### Participation

**Fig 1:**
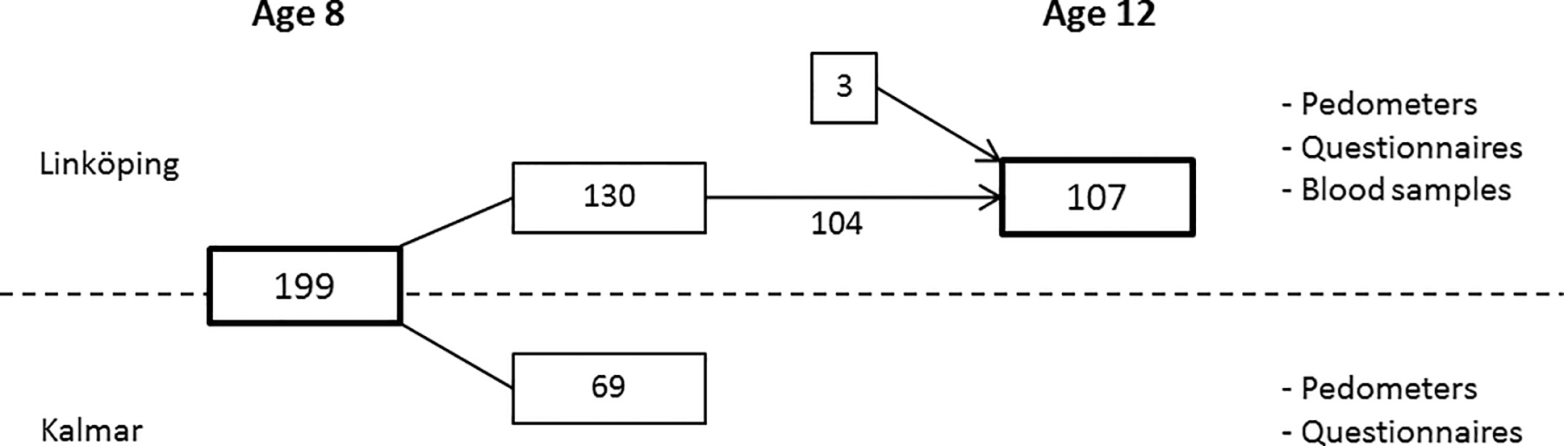
Flow chart describing the participation in the longitudinal follow-up.

Study participation is outlined in Fig 1. All data collection was performed at the schools in Kalmar and Linköping. At age 8, 199 children participated in the assessment (100 girls, 99 boys), 130 from schools in Linköping and 69 from schools in Kalmar. The second assessment had 107 participants (51 girls, 56 boys), this time only from Linköping. Of these 107, 104 belonged to the initial group of 199 children, and in addition 3 class-fellows who absolutely wanted to participate. At age 8, 154 of the parents answered the questionnaire, while 90 parents and 90 children answered the questionnaire at 12 years.

### Physical activity—pedometers

Daily step counts were measured with cable tie sealed Yamax pedometers (SW-200 Tokyo, Japan) during four consecutive weekdays, a time span shown to produce reliable results and avoid reactivity [20]. Pedometers were attached to the waistband and placed in line with the midpoint of the right knee. Participants were instructed to wear the pedometer during the entire day until bedtime and to put it on again immediately after dressing in the morning. During the four days of the 8-year assessment, a research assistant collected the pedometers with a 24 hour interval, unsealed them, documented the number of steps, reset, resealed and returned the devices. At the same time, the children were asked to complete a brief survey (i.e a Swedish version of the Tudor Locke pedometer log [21] to verify that the pedometers were worn according to instructions on the previous day. Survey results were used to identify non-compliant participants, who reported removing their pedometer for ≥ 1h; their data were deleted before analysis and substituted with the individual’s daily mean. If more than one day was non-compliant the individual’s step-data was excluded. In the 12-year assessment, the sealed pedometers were worn for four consecutive weekdays without daily reading. The pedometers were collected and read on the morning of the fifth day, and the total number of steps was divided by the number of days the pedometer was worn. At data collection, each child was interviewed by a research assistant, according to the questions in the survey, asking whether the child had forgot to wear the pedometer at any occasion or had been ill at any time from Monday to Friday. If the child had forgotten to wear the pedometer for a full day, the total amount of steps was divided by the actual number of days the pedometer was worn. Step-data from individuals reporting illness was excluded.

### Physical activity—questionnaires

In questionnaires filled in by parents at 8 years, 4 items relating to the child’s daily level of physical activity were used: 1) hours spent in front of TV/Video/DVD 2) hours spent in front of computer 3) hours spent in physical activity (playing/running around) 4) hours spent reading/doing homework. Each question required a separate answer for weekdays and weekends, and the scale used for the answers was 1 = 0–15 minutes, 2 = 1/2 hour, 3 = 1 hour, 4 = 2 hours, 5 = 3 hours, 6 = 4 hours, 7 = 5 hours, 8 = 6 hours, 9 = 7 hours or more. There was also a question regarding time spent in a car weekly, with the scale 1 = 0–30 minutes, 2 = 1 hour, 3 = 2 hour, 4 = 3 hours, 5 = 4 hours, 6 = 5–6 hours, 7 = 7–8 hours, 8 = 9–10 hours, 9 = 11 hours or more.

In the questionnaire at age 12, parents were again asked to estimate their child’s time spent in physical activity (playing/running around) on weekdays and weekends, and weekly hours spent in a car. The questions concerning hours spent in front of TV/Video/DVD, hours spent in front of a computer and hours spent reading/doing homework were instead asked to the children, still divided into separate answers for weekdays and weekends.

### Anthropometric measures

In the assessment at age 8, measures of height, weight and waist circumference were collected the week before data collection. Height was measured on a wall-attached tape (Friedrich Richter; Kirchenlaibach, Germany), rounded down to the nearest centimeter. Weight was measured on step-up scales (EKS International, Wittiesheim, France, HEFA Digital AB Halmstad, Sweden), recorded with one decimal point. Height and weight were used to calculate BMI. Cut-offs for overweight and obesity were defined according to Cole et al [22], adjusted for age and gender. Measuring tape was used for waist circumference, rounded down to the nearest centimeter. At 12 years, the measurements were performed at the same day as the blood draw, which was performed around the time of data collection (±1 week). Height and weight were measured on step-up scales and wall-attached tapes at the local school nurse offices, recorded as above. Waist circumference was measured as at 8 years, and overweight/obesity cut-offs were defined in the same way.

### Insulin sensitivity—blood glucose control

At 8 years, fasting blood was drawn by research nurses during the same week as measurement of physical activity. Plasma glucose, C-peptide and HbA1c were determined, and HOMA2-IR (HOMA = homeostasis model assessment, IR = insulin resistance) and HOMA2-%B (B = β-cell function) index was calculated [23]. Plasma glucose was measured by the use of One Touch® Ultra (Life Scan, Sweden). HbA1c was measured with an immunological method, calibrated against the Swedish Standard Mono-S and continuously controlled against the Swedish EQUALIS reference method (External Quality Assurance in Laboratory medicine in Sweden). Serum C-peptide was chosen to give a measure of β-cell function, as it is secreted by β-cells in equimolar amounts to insulin, but in contrast to insulin C-peptide passes the liver and gives valid concentrations in peripheral blood. It was measured with a time-resolved fluoroimmunoassay (AutoDELFIA™ C-peptide kit, Wallac, Turku, Finland), as described elsewhere [24].

At age 12, blood draws were performed by research- and school nurses, around the time when pedometers were worn (±1 week). Fasting plasma glucose was measured using Bayer´s BREEZE®2 (Bayer, Sweden). The measurement was performed on venous blood samples, producing 7–9% higher values compared to capillary blood, so the results were adjusted accordingly. C-peptide was measured by the same method as for 8 years, and HbA1c was omitted.

### Statistics

IBM SPSS Statistics 20 was used for statistical analyses. Normality of data distributions was assessed for all objectively measured variables, by D'Agostino & Pearson omnibus normality test and visual inspection of plotted data. Since some variables deviated from Gaussian distribution, non-parametric tests (Spearman correlation and Wilcoxon signed rank test for paired analyses) were performed for statistical analyses performed separately in boys and girls because of smaller group sizes. Correlation analyses performed on the entire group were performed with parametric tests (Pearson correlation). P-values <0.05 were considered statistically significant. Questionnaire data was processed by non-parametric tests (Spearman correlation) because of its ordinal nature. The threshold of significance was lowered to p<0.01 to compensate for multiple comparisons when correlating questionnaire data with objectively measured variables. No such compensation was made for comparisons of only objectively measured data, since the measurements were repeated in the same individuals on two different time points. Linear regression analyses were performed with Fasting C-peptide (nmol/l) and BMI Z-score as dependent variables at age 8 and 12.

## Results

Table 2 describes group characteristics at age 8 and 12, divided by sex. Boys were physically more active than girls at both time points (average daily steps of girls was 89% of the boys mean value at 8 years, and 80% at 12 years). Beside average daily steps, there were no apparent differences between boys and girls, but the rather large difference in physical activity motivated analyses separated for sex, in addition to analyses performed on the group as a whole.

**Table 2:** Group characteristics at age 8 and 12, comparisons between boys and girls, and paired comparisons over time.

	Sex	Age 8	Age 12	Wilcoxon signed-rank test 8 *vs* 12 years	
		Mean (n)	Mean (n)	*n*	*p*
**Steps per day**	*-Girls*	15223 (100)	12853 (50)	50	<0.001
	*-Boys*	17067 (92)	16154 (51)	45	*n*.*s*.
Boys vs girls, p		<0.001	<0.001		
**Body mass index (kg/m**^**2**^**)**	*-Girls*	16.5 (83)	18.6 (49)	49	<0.001
	*-Boys*	16.6 (85)	18.8 (55)	52	<0.001
Boys vs girls, p		*n*.*s*.	*n*.*s*.		
**Waist circumference (cm)**	*-Girls*	59.5 (68)	71.6 (49)	49	<0.001
	*-Boys*	60 (62)	71.1 (55)	52	<0.001
Boys vs girls, p		*n*.*s*.	*n*.*s*.		
**Overweight/obese (%)**	*-Girls*	20 (80)	22.9 (48)	48	*n*.*s*.^*b*^
	*-Boys*	14.5 (83)	20 (55)	50	*n*.*s*.^*b*^
Boys vs girls, p		*n*.*s*.^*a*^	*n*.*s*.^*a*^		
**Fasting C-peptide (nmol/l)**	*-Girls*	0.37 (52)	0.5 (43)	35	0.007
	*-Boys*	0.34 (47)	0.43 (50)	39	0.004
Boys vs girls, p		*n*.*s*.	*n*.*s*.		
**HOMA2-IR**	*-Girls*	0.79 (47)	1.15 (43)	31	0.002
	*-Boys*	0.74 (45)	0.98 (50)	38	0.001
Boys vs girls, p		*n*.*s*.	*n*.*s*.		
**HOMA2-%B**	*-Girls*	96.5 (47)	82.1 (43)	31	*n*.*s*.
	*-Boys*	86.5 (45)	72.5 (50)	38	*n*.*s*.
Boys vs girls, p		*n*.*s*.	*n*.*s*.		
**F-glucose (mmol/l)**	*-Girls*	4.6 (60)	5.1 (45)	41	<0.001
	*-Boys*	4.7 (54)	5.1 (51)	46	<0.001
Boys vs girls, p		*n*.*s*.	*n*.*s*.		
**HbA1c (Mono-S %)**	*-Girls*	3.79 (58)	N/A		N/A
	*-Boys*	3.78 (56)	N/A		N/A
Boys vs girls, p		*n*.*s*.	N/A		

Boys and girls compared by Mann-Whitney U-test n.s. = not significant, N/A = not available

^a^ proportions compared by Fisher´s exact test

^b^ paired proportions compared by McNemar test

### Analysis of objectively measured data: entire group

**Fig 2:**
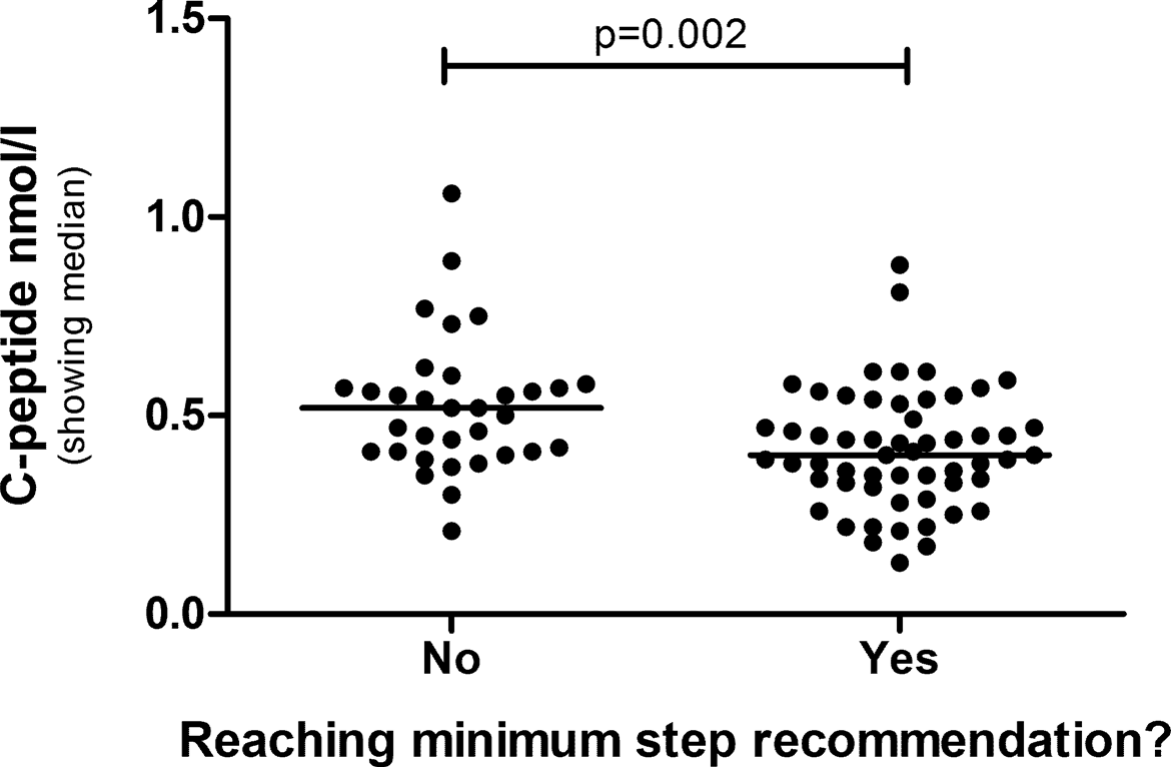
C-peptide level in children reaching the minimum recommendation of daily steps, or not, at age 12. The groups are compared by Mann-Whitney U-test.

A correlation matrix revealed that a large number of variables were significantly correlated to average number of daily steps at age 8. Each of these correlations were confirmed at age 12, with increased statistical significance and higher degrees of correlation (Table 3). When dividing the children into groups based on whether they reached the recommended minimum amount of daily steps or not (BMI-referenced step recommendations for children, 12.000 steps per day for girls and 15.000 steps for boys, [25]), it was shown at age 12 that children taking the recommended amount of steps had lower C-peptide (Fig 2), HOMA2-IR (p = 0.002), HOMA2-%B (p = 0.03) and waist circumference (p = 0.04), compared to children taking too few steps.

**Table 3:** Variables correlated to the average number of daily steps.

	Age 8		Age 12	
	Pearson r	p	Pearson r	p
**BMI**	-.185	0.019	-.232	0.021
**Waist circumference**	-.212	0.018	-.328	0.001
**C-peptide**	-.224	0.029	-.401	<0.001
**HOMA2-IR**	-.236	0.026	-.404	<0.001
**HOMA2-%B**	-.238	0.025	-.271	0.01

As expected, BMI was highly correlated to waist circumference at both time points, but also to HOMA2-IR at 8 and 12 years, C-peptide at12 years and HOMA2-%B at 12 years (Table 4).

**Table 4:** Variables correlated to BMI, other than average daily steps.

	Age 8		Age 12	
	Pearson r	p	Pearson r	p
**Waist circumference**	.797	<0.001	.857	<0.001
**HOMA2-IR**	.208	0.046	.335	0.001
**C-peptide**		*n*.*s*	.334	0.001
**HOMA2-%B**		*n*.*s*	.224	0.031

*n*.*s*. = not significant

To further analyse the association between physical activity and metabolic balance, linear regression analysis was performed with fasting C-peptide (nmol/l) as dependent variable at age 8 and 12, with adjustment for sex and BMI. At age 8, children with more steps per day were less likely to have a higher fasting C-peptide (adjusted Beta = -0.02 95% CI = -0.037- -0.003; p = 0.021; Table 5). This was true also for age 12 (adjusted Beta = = -0.01 95% CI = -0.014–0.127; p = 0.034; Table 6). At age 12 children with a higher BMI Z-score had higher fasting C-peptide (adjusted Beta = 0.04 95% CI = -0.005–0.081; p = 0.029; Table 6).

**Table 5:** Linear regression model with Fasting C-peptide (nmol/l) as dependent variable, age 8 (N = 88).

	P	Adjusted Beta	95% CI
**BMI Z-score**	*n*.*s*.	0.05	- 0.004–0.111
**F-glucose (nmol/l)**	*n*.*s*.	-0.07	-0.161–0.029
**Gender (M/F)**	*n*.*s*.	0.01	- 0.085–0.107
Steps per day*	0.021	-0.02	-0.037- -0.003

n.s. = not significant

*Steps per day divided with 1000

**Table 6:** Linear regression model with Fasting C-peptide (nmol/l) as dependent variable, age 12 (N = 88).

	P	Adjusted Beta	95% CI
**BMI Z-score**	0.029	0.04	0.005–0.081
**F-glucose (nmol/l)**	*n*.*s*.	0.04	-0.038–0.118
**Gender (M/F)**	*n*.*s*.	0.05	-0.015–0.120
Steps per day*	0.034	-0.01	-0.014–0.127

*n*.*s*. = not significant

*Steps per day divided with 1000.

### Analysis of objectively measured data: separated for sex

Because of the difference in physical activity between boys and girls, correlation analyses were repeated separated for sex. None of the significant associations to average daily steps seen at age 8 in the entire group remained statistically significant in the separate groups (data not shown). In 8 year old boys however, C-peptide, HOMA2-IR and HOMA2-%B were all correlated to both BMI (rho = .357 p = 0.014; rho = .335 p = 0.025; rho = .309 p = 0.039, respectively) and waist circumference (rho = .407 p = 0.005; rho = .388 p = 0.008; rho = .362 p = 0.015, respectively), and BMI was highly correlated to waist circumference (rho = .801, p<0.001).

At age 12, the average daily steps of boys was clearly associated to C-peptide (rho = -.516, p<0.001), HOMA2-IR (rho = -502, P<0.001) HOMA2-%B (rho = -.327, p = 0.027, and waist circumference (rho = -.403, P = 0.004), and f-glucose was correlated to C-peptide (rho = .368, p = 0.009) and HOMA2-IR (rho = .430, p = 0.002). BMI remained highly correlated to waist circumference (rho = .751, p<0.001).

**Fig 3:**
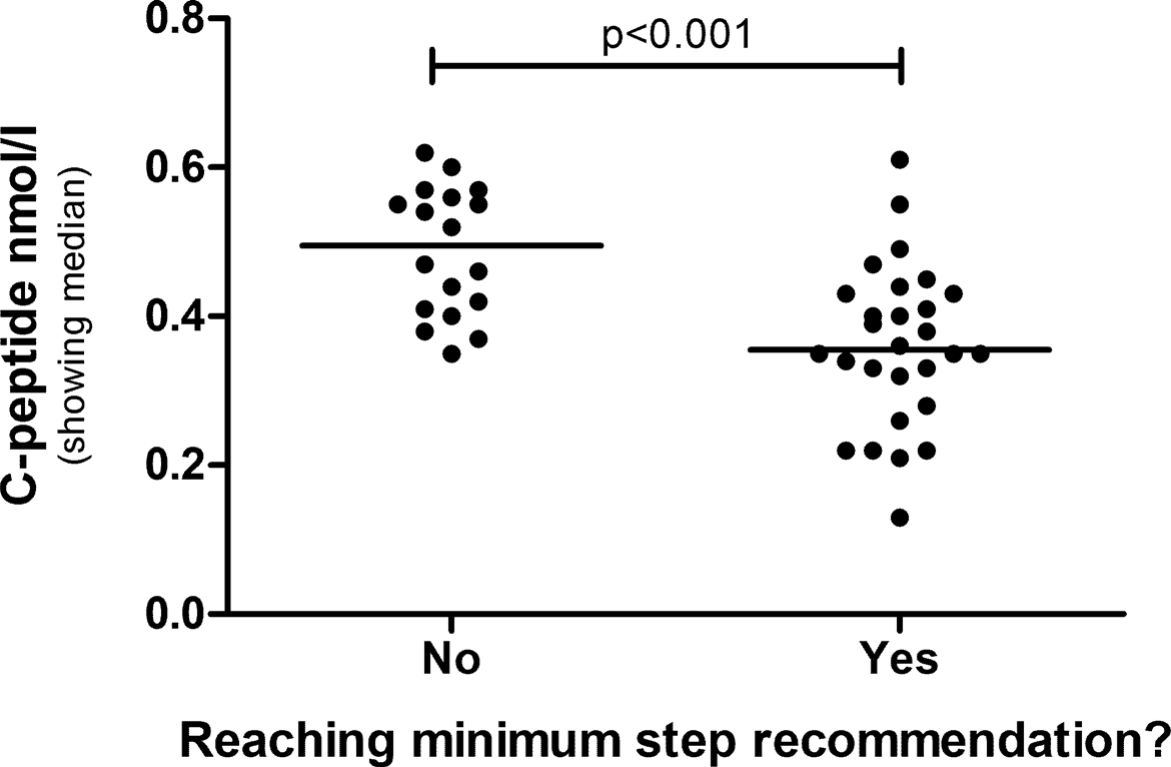
C-peptide level in boys reaching the minimum recommendation of daily steps, or not, at age 12. The groups are compared by Mann-Whitney U-test.

Dividing the boys into groups based on recommended minimum amount of daily steps showed that boys reaching the recommended amount (>15.000 steps) had significantly lower C-peptide (Fig 3), HOMA2-IR (p<0.001), HOMA2-%B (p = 0.013) and waist circumference (p = 0.015), just as seen in the group as a whole.

At age 8, the only significant correlation found in girls was that of BMI and waist circumference (rho = .719, p<0.001). It was noticed that none of the girls with high number of daily steps had high HOMA2-IR, although this could not be proven with statistical significance. At age 12, BMI was still highly correlated to waist circumference (rho = .821, p<0.001), and waist circumference was positively correlated to both C-peptide and HOMA2-IR (rho = .327, p = 0.032). There were no significant correlations between average daily steps and anthropometric measures or laboratory values in girls at age 12 (data not shown). When dividing the girls into groups based on recommended minimum amount of daily steps (>12.000 steps), no differences were seen between girls over and under the cut-off, at either time point (data not shown). That was true also when applying the cut-off used for boys (>15.000 steps).

### Correlation analysis of objectively measured data and questionnaire data

When correlating objectively measured data with questionnaire data across the entire group (boys and girls together), no significant associations were seen at either 8 or 12 years (data not shown). When analyzing the genders separately however, a negative association was revealed between parent-reported daily hours spent outdoors on schooldays and HOMA2-%B in boys at age 8 (rho = -.442, p = 0.004). At age 12, boys had a negative correlation between average daily steps and daily leisure time hours in front of TV/video/DVD on school days reported by the child (rho = -.416, p = 0.006).

### Analyses on longitudinal data

As seen in Table 2, the average number of daily steps decreased significantly with age in girls, while there was no correlation between the girls step values at age 8 and 12 (data not shown). Boys had a non-significant decrease in steps from 8 to 12 years, and the values were significantly correlated (rho = .418, p = 0.004), indicating that the individual level of physical activity was more stable over time in boys. BMI, waist circumference, C-peptide, HOMA2-IR and f-glucose all increased with age in boys and girls (Table 2). BMI at age 8 was highly correlated to BMI at age 12 in both boys and girls (rho = .866, p<0.001 and rho = .815 p<0.001, respectively), and the same stands for waist circumference (rho = .703, p<0.001 and rho = .731, p<0.001, respectively). In boys, there was also a correlation of f-glucose over time (rho = .486, p<0.001).

**Fig 4:**
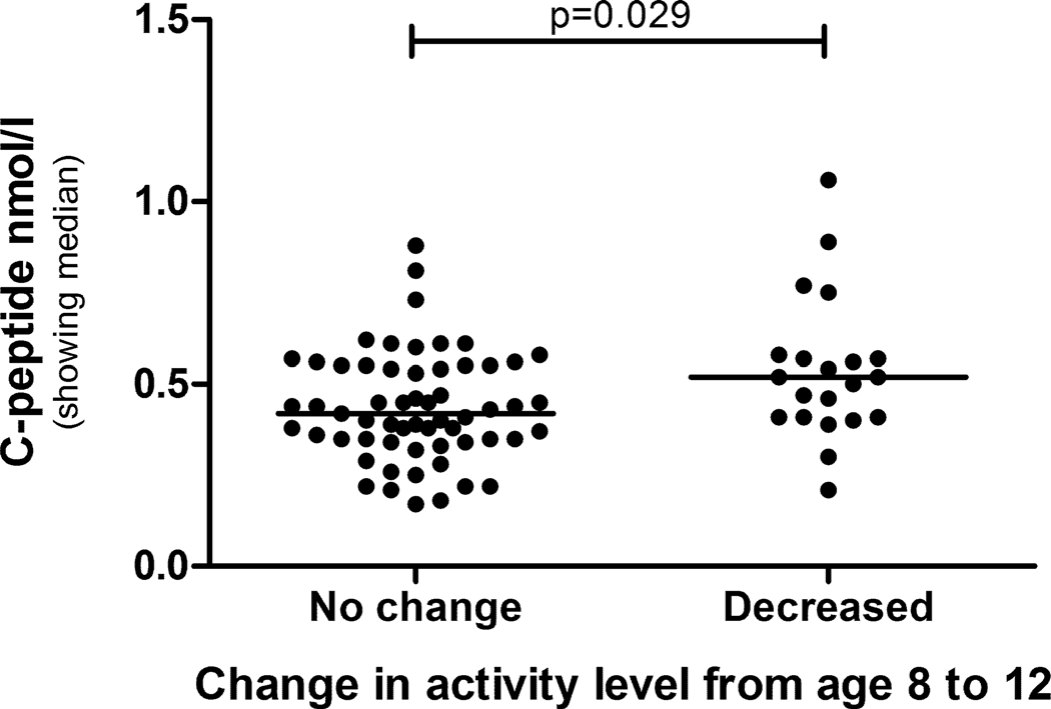
C-peptide level in children that maintained or decreased their physical activity over time. The groups are compared by Mann-Whitney U-test.

**Fig 5:**
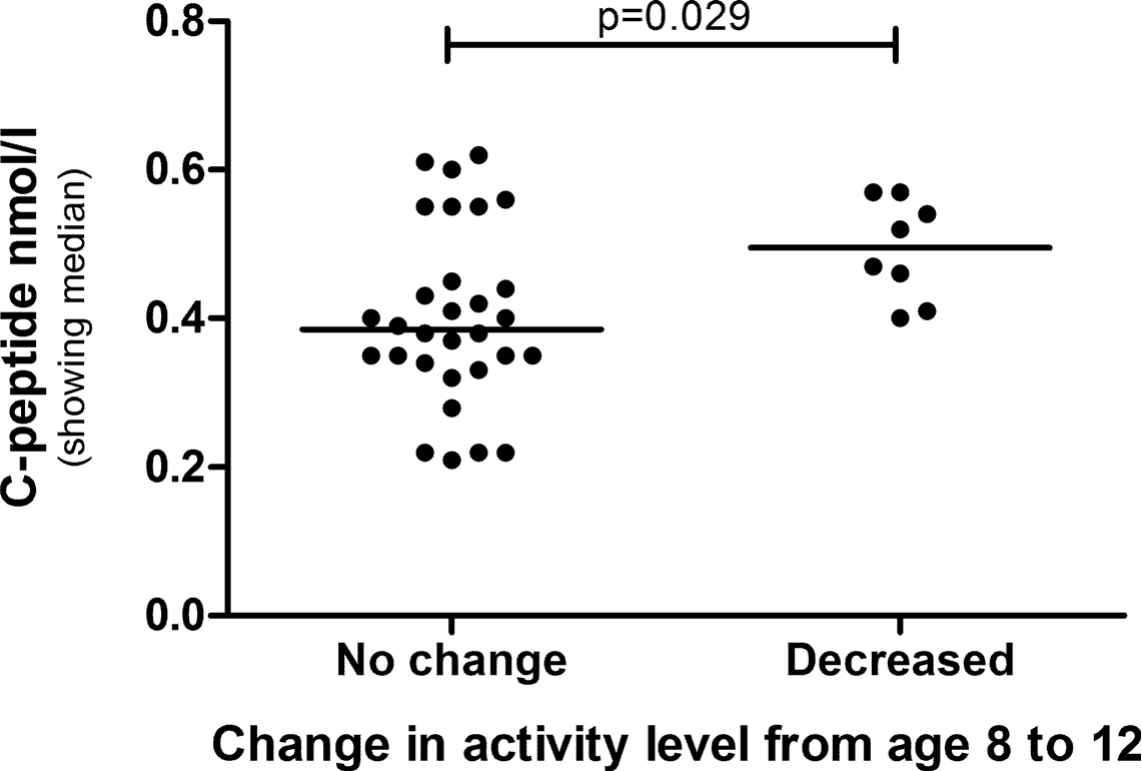
C-peptide level in boys that maintained or decreased their physical activity over time. The groups are compared by Mann-Whitney U-test.

The children were divided into groups based on their change in physical activity from age 8 to 12. Children who decreased their physical activity over time got significantly higher C-peptide (Fig 4) and HOMA2-IR (p = 0.041). When analyzing boys and girls separately, these differences could only be seen in boys (Fig 5), with HOMA2-IR showing borderline significance (p = 0.059).

## Discussion

Our results show that low physical activity, already in school age, is related to increased β-cell load (indicated by elevated C-peptide) and insulin resistance. This was seen in the total group of children but especially pronounced in boys, who were more physically active than girls at both time points. The associations seen at 8 years of age were similar at age 12 in most children. Linear regression model shows that children with more steps per day were less likely to have a higher fasting C-peptide and children with a higher BMI Z-Score had higher fasting C-peptide (age 12).

Similar observations have been made before but the longitudinal follow-up of our children is a strength, and shows that the association seen at age 8 in a child tends to remain or even become more pronounced with increasing age. Children decreasing their physical activity get significantly higher C-peptide and increased insulin resistance measured as HOMA index. The findings are most pronounced in boys. When the analyses were performed on boys and girls separately, the differences seen at age 8 were no longer statistically significant, probably due to the smaller group sizes. At age 12 however, several parameters were significantly correlated to daily steps in boys (C-peptide, HOMA2-%B, HOMA2-IR, waist circumference), while no significant correlations were seen in girls. Some girls were on their way into puberty at age 12 rendering them more insulin resistant. This might explain why effects of physical activity were more difficult to see in the girls. In addition, girls repeatedly showed a more narrow range of daily steps and lower levels of physical activity than boys, and it could be speculated that a certain threshold of activity needs to be reached before metabolic benefits are gained, in particular during puberty. The difference in physical activity between boys and girls has been shown before [26], and it highlights the importance of promoting physical activity in school age, not least in girls.

Our results support and strengthen previous studies showing that a sedentary life style is associated with cardio-metabolic risk [27]. Evidence for an association between sedentary behavior and insulin concentration has been found, while an association between sedentary behavior and inflammatory markers has not been clearly shown [28]. C-peptide levels increased significantly over time in both boys and girls, in agreement with other studies [29]. Girls decreased their amount of daily steps significantly with increasing age, also seen in boys, but with no statistical significance in accordance with international research [30].

BMI and waist circumference seemed stable over time. Children with high BMI at age 8 tended to have high BMI also at age 12 [31]. BMI was correlated to insulin resistance and C-peptide, at both 8 and 12 years, most pronounced in boys. The associations between physical activity and anthropometric measures were weak, as seen in previous studies [32,33]. However, physical activity had a stronger impact on insulin resistance/β-cells stress. An Australian study also found an effect of physical activity on insulin resistance [34], but only in boys.

Physical activity and its effect on insulin resistance is known to be important for development of type 2 diabetes, but might also play a role for development of type 1 diabetes. According to the β-cell stress hypothesis overweight, rapid weight gain and low physical activity may contribute to β-cell stress or overload, leading not only to increased insulin secretion but also to increased presentation of pancreatic autoantigens [35]. This could in turn increase the risk of autoimmune reactions directed towards islets and development of T1D in genetically predisposed children. Thus, our modern, sedentary life style might already in low ages contribute to the increasing incidence of T1D in children.

Reliable measurement of physical activity is difficult. Subjective measurement (self-report or parent proxy-report for smaller children) of physical activity/sedentary behavior is commonly used because it is simple and cost effective, but such methods often suffer from poor validity/reliability, and high variability due to subject bias and difficulties in accurate recalling [36,37]. In our study, parents and children answered questions regarding physical activity/sedentary behavior as a complement to objective measurement by pedometers. Since our expression of physical activity is mean steps per day, one could argue whether increased leg length and consequently stride length could result in a decline of accumulated steps. Stride length was not measured in this study, but an earlier study with the same measurement instrument and procedure (i.e height as a substitute for leg length) has examined the importance of stride length [38]. The energy cost of a step is reported as being economically equal whether taken by a small or large individual [39]. Taken at the same speed, the number of steps taken can be viewed equivalent. Some consistency was seen in boys between subjectively estimated TV-time and objectively measured physical activity, and time spent outdoors was negatively correlated to HOMA2-%B. Although the relations between subjective and objective variables are sporadic and only seen in boys, they do point in the expected directions. Motion sensors, like pedometers, can cost-effectively produce valid and reliable activity data in an unobtrusive, convenient, unbiased way [20]. To control for wear time we used an assurance survey considered as standard procedure in pedometry. The use of accelerometers with the capacity to time stamp wear time might have been an alternative. However, the lack of standardization in collecting and processing accelerometer data, such as different cut-points, epoch times and model changes over time [40] is a burden when comparing cross-sectional samples over time or in longitudinal studies. Another advantage of pedometers is the possibility to communicate physical activity in an understandable way, as steps per day recommendations to parents, teachers and health professionals, thereby providing support in intervention and surveillance [41]. In our study, objective measurement appears reliable, and both body measures and laboratory values show better agreement to pedometer steps than to questionnaire data.

## Conclusion

In conclusion, physical activity is related to insulin resistance and β-cell stress, and a decrease in physical activity already in school-age is associated with increased insulin resistance and load on the insulin producing β-cells, which might be of importance for development of both type 1- and 2 diabetes. Physical activity should be promoted for school children.
